# Caveolin-3 Microdomain: Arrhythmia Implications for Potassium Inward Rectifier and Cardiac Sodium Channel

**DOI:** 10.3389/fphys.2018.01548

**Published:** 2018-11-09

**Authors:** Ravi Vaidyanathan, Louise Reilly, Lee L. Eckhardt

**Affiliations:** Cellular and Molecular Arrhythmia Research Program, University of Wisconsin-Madison, Madison, WI, United States

**Keywords:** potassium channel, potassium channel (inward-rectifier, outward-rectifier), Cav3, microdomain, sodium channel

## Abstract

In human cardiac ventricular myocytes, caveolin-3 functions as a scaffolding and regulatory protein for signaling molecules and compartmentalizes ion channels. Our lab has recently explored this sub-cellular microdomain and found that potassium inward rectifier Kir2.x is found in association with caveolin-3. The three cardiac Kir2.x isoforms (Kir2.1, Kir2.2, and Kir2.3) are the molecular correlates of I_K1_ in the heart, of which Kir2.1 is the dominant isoform in the ventricle. Kir2.1 channels assemble with Kir2.2 and Kir2.3 forming hetero-tetramers that modulate I_K1_. I_K1_ sets the resting membrane potential and assists with terminal phase 3 ventricular repolarization. In our studies using native human ventricular tissue, Kir2.x co-localizes with caveolin-3 and significance of the association between Kir2.x and caveolin-3 is emphasized in relation to mutations in the gene which encodes caveolin-3, *CAV3*, associated with Long QT Syndrome 9 (LQT9). LQT9-associated *CAV3* mutations cause decreased current density in Kir2.1 and Kir2.2 as homomeric and heteromeric channels, which affects repolarization and membrane potential stability. A portion of Kir2.1 cardiac localization parallels that of the cardiac sodium channel (Nav1.5). This may have implications for Long QT9 in which *CAV3* mutations cause an increase in the late current of Nav1.5 (I_Na−L_) via nNOS mediated nitrosylation of Nav1.5. In iPS-CMs, expression of LQT9 *CAV3* mutations resulted in action potential duration (APD) prolongation and early-after depolarizations (EADs), supporting the arrhythmogenicity of LQT9. To evaluate the combined effect of the *CAV3* mutants on I_Na−L_ and I_K1_, we studied both ventricular and Purkinje myocyte mathematical modeling. Interestingly, mathematical ventricular myocytes, similar to iPS-CMs, demonstrated EADs but no sustained arrhythmia. In contrast, Purkinje modeling demonstrated delayed-after depolarizations (DADs) driven mechanism for sustained arrhythmia, dependent on the combined loss of I_K1_ and gain of I_Na−L_. This finding changes the overall assumed arrhythmia phenotype for LQT9. In future studies, we are exploring caveolar micro-domain disruption in heart failure and how this effects Kir2.x and Nav1.5. Here we review the caveolae cardiac microdomain of Kir2.x and Nav1.5 and explore some of the downstream effects of caveolin-3 and caveolae disruption in specific clinical scenarios.

## Caveolin and Ion Channel Microdomains

Caveolae (Latin for ‘little caves’) are small (50–100 nm) structural invaginations in the lipid bilayer enriched in sphingolipids, cholesterol, and created by oligiomerized scafolding protein caveolin (Bastiani and Parton, 2010). The caveolin family of proteins is encoded by 3 genes (*CAV1*, *CAV2*, and *CAV3*) and consists of six known caveolin subtypes: caveolin-1a and 1b, caveolin-2α, 2β and 2γ, and caveolin-3 (Cav3) (Balijepalli and Kamp, 2008). Cav3 is specifically expressed in muscle tissue including the heart, where it functions as a scaffolding protein and assembles signaling complexes that can regulate the function of ion channels in caveolae. Caveolae have been shown to be present in atria, ventricle and nodal cells in the heart (Balijepalli and Kamp, 2008). Multiple ion channels and transporters expressed in the heart such as the L-Type calcium channel (Cav1.2) (Balijepalli et al., 2006), T-type calcium channel (Cav3.1) (Markandeya et al., 2011), sodium channel (Nav1.5) (Yarbrough et al., 2002), potassium channels including the inward rectifier potassium channel (Kir2.x) (Vaidyanathan et al., 2013), pacemaker channel (HCN4) (Ye et al., 2008), the sodium/calcium exchanger (NCX1) (Bossuyt et al., 2002) and others have been shown to localize to caveolae. Mutations in Cav3 disrupt these signaling and coordinating microdomains and can cause structural cardiac and arrhythmic disease such as long QT syndrome (LQT9), sudden infant death syndrome, and hypertrophic cardiomyopathy (Hayashi et al., 2004; Vatta et al., 2006; Cronk et al., 2007).

This review will focus on the importance of this microdomain and how it determines cardiac excitability. Recent publications by us and other groups have reported that the ion channels that regulate cardiac excitability form membrane bound macromolecular complexes (Milstein et al., 2012; Vaidyanathan et al., 2013, 2018). Additionally, we will discuss the downstream effects of Cav3 mutations on cardiac excitability as a cause for ventricular arrhythmias in inherited arrhythmia syndromes.

## Ion Channels Involved in Cardiac Excitability

The cardiac action potential is determined by an interplay of *trans*-membrane ionic currents in myocardial cells. Depolarization of cellular membranes in atrial, ventricular and Purkinje cells is dominated by cardiac sodium (Na^+^) current (I_Na_) through voltage-gated sodium channels, Nav1.5. Depolarization from −80 to −70 mV to peak voltages of +30 to +40 mV allows for activation of the L-type calcium (Ca^2+^) channels and induction of calcium-induced-calcium-release to facilitate excitation-contraction coupling. Repolarization is a complex process governed by the gradual activation of outward potassium (K^+^) currents and inactivation of depolarizing inward currents (Na^+^ and L-type Ca^2+^ channels). The terminal phases of cellular repolarization to the resting membrane current depends on the inward rectifying K^+^ current (I_K1_) and the molecular correlates of I_K1_ are the Kir2.x family (Kir2.1, Kir2.2, and Kir2.3) of ion channels. For the next action potential to be initiated, channels carrying depolarizing current (usually Na^+^ channels) must recover to a closed state before they can reopen for the next action potential. Na^+^ channel recovery occurs when the cell is polarized by I_K1_. Thus, by controlling the resting membrane potential, I_K1_ modifies sodium channel availability and therefore I_Na_, cell excitability, action potential duration, and velocity of impulse propagation.

## Association of Kir2.x and Nav1.5 in Caveolar Complexes

We were the first to identify that Kir2.x channels associate with Cav3 in the heart (Vaidyanathan et al., 2013, 2018). This novel interaction was inspired by the presence of prominent U-waves on electrocardiograms (ECG) from an LQT patient with a *CAV3* mutation. This ECG feature is found in patients with low K^+^ or in genetic conditions with loss of Kir2.1 function, such as Andersen-Tawil Syndrome (Tristani-Firouzi et al., 2002), thus, suggesting that LQT9 associated *CAV3* mutations could possibly affect the function of Kir2.1. We used multiple molecular techniques including immunostaining, co-immunoprecipitation, and fluorescent resonant energy transfer (FRET) to demonstrate the association between Cav3 and Kir2.1 (directly or indirectly). We also identified residues/domains on each protein required for this association. Other cardiac Kir2.x isoforms, Kir2.2 and Kir2.3, associate with Cav3 and the residues of Kir2.x that are crucial for the association with Cav3 appear to be a conserved N-terminal sequence containing a caveolin-binding motif (CBM) composed of a specific sequence of aromatic amino acids including QxQxxxxQ where “Q” is an aromatic amino acid residue (tyrosine, Y; tryptophan, W; and phenylalanine F) and a “x” represents any other residue (Raab-Graham et al., 1994; Couet et al., 1997; Han et al., 2014). We found that the Kir2.x CBM is required for co-immunoprecipitation as deletion of this results in no association of Cav3 and Kir2.x (Vaidyanathan et al., 2018). This finding should be taken into context, because CBM sequences as evidence for a Cav3 interaction site has been questioned, largely related to the relationship of CBM and accessibility based on protein 3D structure (Byrne et al., 2012; Collins et al., 2012). We suspect that the Kir2.x N-terminal CBM (amino acids 81–92) is accessible based on both our findings as well as its close proximity to amino acids which interact with other cytoplasmic regulatory molecules including phosphatidylinositol biphosphate and nitric oxide (Donaldson et al., 2003; Gomez et al., 2009). Cav3 has 4 domains: N-terminal (NT; aa 1–54), scaffolding (aa 55–73), membrane-associated (aa 74–106), and C-terminal (CT; aa 107–151). We also determined that only the scaffolding and membrane domains associate with Kir2.x. The implications of this are that disruption of the Kir2.x CBM sequence or scaffolding or membrane Cav3 domains may affect the presence of Kir2.x channels in caveolar microdomains.

The association of Cav3 and Nav1.5 has been demonstrated by several investigators (Vatta et al., 2006; Cronk et al., 2007; Cheng et al., 2013). Na_V_1.5 localizes to caveolin-rich membrane domains, demonstrated by co-immunoprecipitation in heterologous cells (HEK293 cells) but also in rat ventricular tissue (Yarbrough et al., 2002; Vatta et al., 2006). It is currently unclear if the interaction of Cav3 with Nav1.5 is direct or indirect. However, in rat ventricular myocytes these proteins appear to be part of a macromolecular complex composed of syntrophin alpha-1, neuronal nitric oxide synthase (nNOS), Cav3, and Nav1.5 (Cheng et al., 2013).

**FIGURE 1:**
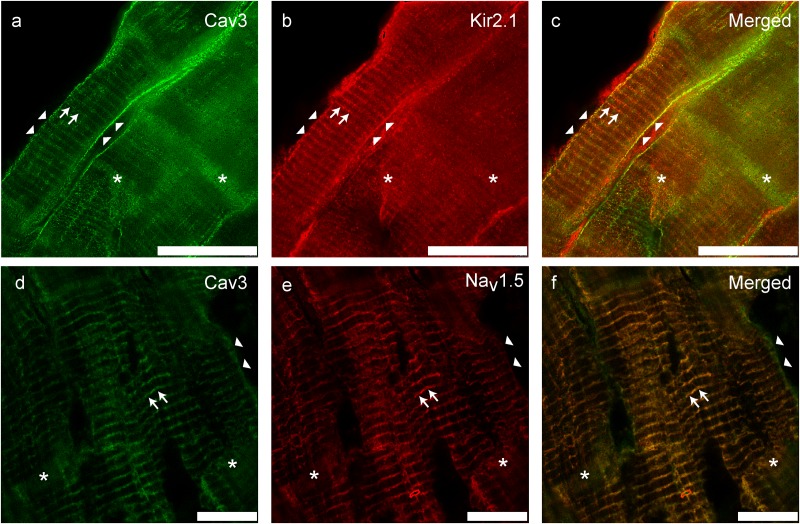
Immuno-colocalization of Kir2.1 **(b)** and Nav1.5 **(e)** with Cav3 **(a,d)** in human ventricular tissue (adapted from Vaidyanathan et al., 2018). **(c,f)** Represent merged images; yellow color indicates areas of overlap. Scale bar = 25 μm.

We previously investigated and reported the association of Kir2.x and Cav3 in human ventricular cardiomyocytes (Vaidyanathan et al., 2018). We demonstrated by employing stimulated emission depletion (STED) microscopy, a super-resolution microscopy technique, Kir2.1 localized to the T-tubules, lateral membrane and intercalated disk in cardiomyocytes. Others have reported that Kir2.1 and Nav1.5 channels are not only part of the same macromolecular complex in cardiac myocytes (Milstein et al., 2012; Matamoros et al., 2016) but also traffic together (Ponce-Balbuena et al., 2018). As shown in Figure 1, Nav1.5, Kir2.1, and Cav3 co-localize to similar sub-cellular locations suggesting that they are part of the same macromolecular complex (Vaidyanathan et al., 2018). Kir2.x isoforms localize to different regions: Kir2.1 localizes to the sarcolemma, T-tubules and intercalated disk, Kir2.2 preferentially is found at the T-tubules, and Kir2.3 localizes to the intercalated disk. However, Kir2.1 and Nav1.5 immunolocalize with Cav3 with a Pearson’s correlation coefficient >0.5, suggesting close localization of Kir2.1 with Cav3 and Nav1.5 with Cav3.

## Arrhythmia Pathology Related to Abnormal Cav3

Mutations in *CAV3* cause several types of muscle related clinical diseases including muscular dystrophy, hypertrophic cardiomyopathy and the arrhythmia syndrome of Long QT syndrome (LQTS) (Balijepalli and Kamp, 2008). From a cohort of patients referred for genetic testing for LQTS, several *CAV3* mutations were identified from individuals who were gene negative for other known LQTS genes [*KCNQ1* (LQT1), *KCNH2* (LQT2), *SCN5A* (LQT3), *KCNE1* (LQT5), *KCNE2* (LQT6), and *KCNJ2* (ATS1) and targeted analysis of *ANK2* (LQT4) and *RyR2* (CPVT1)] (Vatta et al., 2006). To determine how these *CAV3* mutations cause LQTS, constructs of the mutations were expressed in cells with ion channels known to associate with Cav3. When co-expressed with Nav1.5, LQT-associated *CAV3* mutations did not affect the peak current density of I_Na_ compared to wild type (WT). However, F97C-Cav3 and S141R-Cav3 increased late I_Na_ (I_Na-L_) by ∼2-3 fold compared to vector and WT-Cav3. In the complex containing Nav1.5, Cav3, alpha-1-syntrophin, and nNOS, Cav3 inhibits nNOS mediated nitrosylation of Nav1.5 (Cheng et al., 2013). However, LQT9 associated mutation F97C-Cav3 remains in the complex but has lost the ability to suppress nNOS mediated nitrosylation of Nav1.5. Thus, it appeared that I_Na-L_ increases due to nitrosylation of Nav1.5 when LQT9 mutations are present.

**FIGURE 2:**
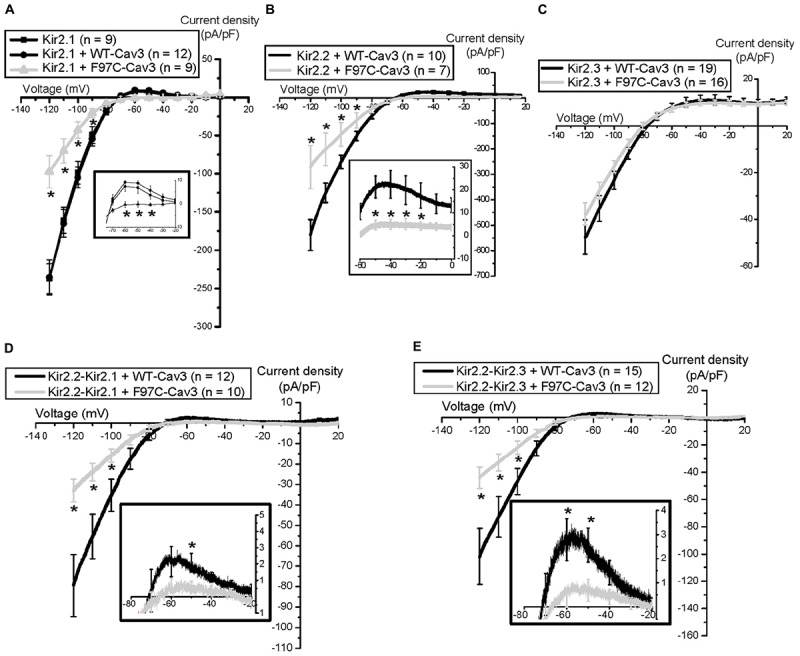
Electrophysiological properties of Nav1.5 channels and Kir2.x homomeric and heteromeric channels with F97C-Cav3 (Vaidyanathan et al., 2018). ^∗^*p* < 0.05.

We observed that one patient with LQT9 also had prominent U-waves (Vatta et al., 2006), which is an electrocardiographic feature seen in patients with loss of function mutations in the Kir2.1 related to Andersen-Tawil Syndrome (ATS1) (Tristani-Firouzi et al., 2002). For these reasons, we investigated the effect of LQT9 mutations on Kir2.x channels by co-expressing homomeric Kir2.1 or Kir2.2 or Kir2.3 with WT-Cav3 or LQT9-associated F97C-Cav3 (Vaidyanathan et al., 2013, 2018). Interestingly, F97C-Cav3 decreased peak inward and outward current density of homomeric Kir2.1 (Figure 2A) and homomeric Kir2.2 (Figure 2B) by ∼ 50–60% but not homomeric Kir2.3 (Figure 2C) compared to WT-Cav3. Since Kir2.x channels can be present as heteromeric channels in cardiomyocytes and given the differential effect of F97C-Cav3 on Kir2.x channels, we created heteromeric vectors of Kir2.2-P2A-Kir2.1 and Kir2.2-P2A-Kir2.3 (P2A is a self-cleaving peptide). When heteromeric channels are co-expressed with F97C-Cav3, peak inward and outward current density decreased compared to WT-Cav3 (Figures 2D,E). Interestingly, even though F97C-Cav3 had no effect on Kir2.3 homomeric channels, there is decreased peak inward and outward current of the Kir2.2-Kir2.3 heteromeric channels compared to WT-Cav3, suggesting that Cav3 is able to regulate Kir2.3 when expressed with Kir2.2. Perhaps this is related to channel assembly leading to lack of membrane expression as we determined that the F97C-Cav3 mutation caused a 50% reduction in membrane trafficking of Kir2.1 and Kir2.2 channels (Vaidyanathan et al., 2013, 2018). Immunostaining experiments suggested that Kir2.1 channels are localized to the Golgi when co-expressed with F97C-Cav3, which is the site for channel tetrameric assembly. Thus, it is possible that the F97C-Cav3 prohibits heteromeric channels from leaving the Golgi and decreases current density, decreasing I_K1_ and membrane repolarization.

## Dual Cellular Molecular Mechanism of *Cav3* Mutations Causing LQT

**FIGURE 3:**
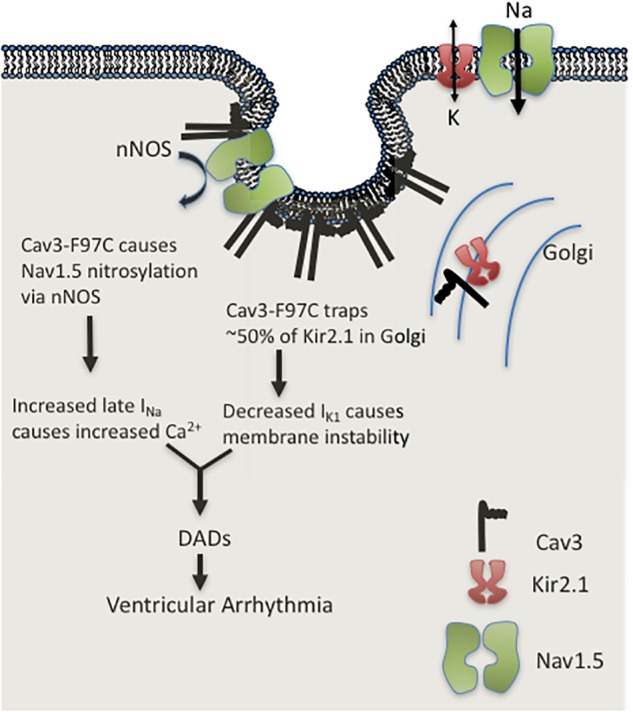
Cartoon illustration of LQT9 arrhythmia mechanism.

The initial studies demonstrating the major effects of LQT9 mutations left significant questions as there are two distinct cellular mechanisms causing ionic current abnormalities: gain of I_*Na-L*_ due to sodium channel nitrosylation and inhibition of Kir2.1 and Kir2.2 channel membrane trafficking causing loss of I_K1._ How and by what mechanism do LQT9 Cav3 mutations cause a clinical ventricular arrhythmia? Cheng et. al. demonstrated in rat ventricular myocytes in culture, over-expression of WT-Cav3 or F97C-Cav3 caused significant prolongation of action potential duration (APD) at 90% repolarization in cells expressing F97C-Cav3 as compared to cells expressing WT-Cav3. This effect was reversed by the addition of L-NMMA (an nNOS inhibitor). We also demonstrated that in human iPS cardiomyocytes a similar prolongation of APD at 50, 70, and 90% repolarization occurred in cells with F97C-Cav3 compared to WT-Cav3 at 0.5 and 1 Hz pacing frequency (Vaidyanathan et al., 2016). At lower pacing frequency (0.33 Hz) we recorded early after depolarization’s (EADs). EADs are the triggered activity required in congenital or drug-induced LQTS to induce a specific type of polymorphic ventricular tachycardia called torsade de pointes (Vaidyanathan et al., 2016). Thus, we hypothesized that with action potential prolongation due to I_*Na-L*_ and decreased I_K1_, EAD triggered activity is the arrhythmia mechanism in LQT9. To test this, we ran mathematical simulation on a human ventricular cell model (Grandi et al., 2010; O’Hara et al., 2011) in our recent report (Vaidyanathan et al., 2018). In the human ventricular cell model by either decreasing I_K1_ or increasing I_*Na-L*_, the APD prolonged and increased I_*Na-L*_ induced EADs at low pacing frequency. Unexpectedly, when both, increased I_*Na-L*_ and decreased I_K1_ was simulated in the model, EADs were prevented because of resting membrane potential depolarization that decreasing total I_*Na*_ and thus I_*Na-L*_. We then tested same ionic current changes found experimentally in LQT9 using the Li-Rudy canine Purkinje cell model (Li and Rudy, 2011). In contrast to ventricular myocyte model, the Purkinje cell model with increased I_*Na-L*_ and decreased I_K1_ demonstrated delayed after depolarizations (DADs), prominent at both low (0.25 Hz) and high (3.33 Hz) pacing frequencies (Vaidyanathan et al., 2018). At these frequencies, the mechanism involved calcium loading due to increased I_*Na-L*_, and unstable resting membrane potential due to decreased I_K1_ density. As depicted in Figure 3, this increased cellular calcium load combined with reduced repolarization reserve decreased the DAD threshold leading to sustained arrhythmia. Although the cellular ventricular myocytes show APD prolongation and EADs, sustained arrhythmia is triggered from DADs in the Purkinje cells. This finding changes the overall assumed arrhythmia mechanism for LQT9.

## LQT9 Vs. “Classical” LQTs Arrhythmia Mechanisms

The cellular mechanism for LQTS is related to gain or loss of ion channel function directly or indirectly by mutations which reduce ion channel surface membrane expression or by ion channel accessory/regulatory proteins which can exert a direct or indirect effect. While there are currently 17 different LQTS types based on the gene implicated, the most common three LQTS are: LQT1 involving mutations in the *KCNQ1* gene (30–35% of patients), LQT2 involves mutations in the *KCNH2* gene (25–30% of patients) and LQT3 involves mutations in the *SCN5a* gene (10% of patients) (Cerrone et al., 2012; Priori et al., 2013). LQT4-17 are more rare, including LQT9 and based on our work and others, the mechanism of arrhythmia initiation may be dissimilar to classically described LQTS (Koenig and Mohler, 2017; Vaidyanathan et al., 2018). The signature arrhythmia in LQTS is torsade de pointes, resulting from decreased repolarization reserve and susceptibility for EADs (Marban et al., 1986). EADs occur when the action potential duration is prolonged, which permits more time for the time- and voltage-dependent recovery and reopening of L-type Ca^2+^ channels at plateau voltages to carry added depolarizing current (January et al., 1988). In LQT9, we did not find that EAD initiation led to sustained arrhythmia, but rather purkinje cell DADs causes sustained arrhythmia (Vaidyanathan et al., 2018). DADs and EADs are different mechanistically. DADs result from the overloading of cells with Ca^2+^, which then overloads the sarcoplasmic reticulum (SR) with Ca^2+^. The SR releases Ca^2+^ during an action potential and under conditions of Ca^2+^ overload is transiently re-released into the myoplasm. These transient rises in myoplasmic Ca^2+^ activate Ca^2+^–dependent depolarizing membrane current, mostly through the Na^+^ – Ca^2+^ exchange causing voltage oscillations or a DADs (Ter Keurs and Boyden, 2007; Fink et al., 2011). DADs that reach voltage threshold can initiate a subsequent action potential. These divergent mechanisms for triggered activity are not only distinct experimentally, they are approached differently clinically, as DADs are enhanced by rapid stimulation, whereas EADs occur at slow stimulation rates where action potential duration is longest and are usually abolished at higher stimulation rates. Our model of LQT9 is speculative but is akin to Purkinje-dependent DAD perpetuation in EAD-susceptible myocytes in heart failure (Myles et al., 2012). This highlights the overall impact of our investigation and the importance of ongoing studies to optimize treatment approaches for these arrhythmia syndromes.

## Cav3 in Heart Failure and Downstream Microdomain Dysregulation

Beyond more rare *CAV3* mutations, Cav3 is also down regulated in the ventricle of animal models of heart failure and in human heart failure (Feiner et al., 2011). Multiple murine models of heart failure such as pressure overload induced by transverse aortic constriction (TAC) (Feiner et al., 2011), transgenic mice with constitutive overexpression of A1-adenosine receptor and angiotensin-II infusion (Markandeya et al., 2015), report that there is significant loss of Cav3 and caveolae at the sarcolemma in ventricular myocytes. Woodman et. al. report that Cav3 knockout (KO) mice develop progressive cardiomyopathy at 4 months of age marked by significant hypertrophy, dilation, and reduced fractional shortening (Woodman et al., 2002). Interestingly, over-expression (OE) of Cav3 in mice attenuates hypertrophy phenotype (Horikawa et al., 2011). When Cav3 OE mice are exposed to TAC, they had increased survival, reduced cardiac hypertrophy and preservation of cardiac function as compared to control mice (Horikawa et al., 2011).

Ion channel remodeling occurs in animal models of heart failure. L-type calcium channel current (I_Ca-L_) is decreased in heart failure has in part been attributed to loss of Cav3 (Bryant et al., 2018a,b). Bryant et al. (2018a) report that in TAC exposed mice, there was T-tubule disruption, decreased expression of Junctophilin 2 and Cav3, impairment of calcium release at the T-tubules and decreased I_Ca-L_ at the T-tubule with no change in I_Ca-L_ at the sarcolemma. These results suggest that Cav3 microdomains play a key role in maintaining normal physiology and perturbations of caveolae can cause pathology.

In systolic heart failure it has been reported that I_K1_ and I_Na_ are downregulated (Beuckelmann et al., 1993; Li et al., 2004; Valdivia et al., 2005), however, it is not yet understood if this is related to the loss of caveolae, as with L-type calcium channel. We are currently investigating if decreased I_K1_ and I_Na_ in heart failure is related to loss of caveolae or Cav3 regulation. Due to the complexity we have observed of cell type (modeling experiments) and the differences in effects by *CAV3* mutations on I_K1_ and I_Na_, we anticipate that there may also be a complex effect in heart failure on Kir2.x and Nav1.5 remodeling. We hope that answering these questions of caveolar microdomain disruption in various forms of heart failure will lead to improved clarity of ionic channel remodeling in heart failure.

## Conclusion

Cav3 microdomain containing Kir2.x and Nav1.5 in cardiomyocytes are an essential part of normal cardiac physiology. Mutations in *CAV3* cause increased I_Na−L_ and decreased I_K1_ resulting in membrane instability and mathematical modeling suggests this causes calcium loading leading to DAD-dependent arrhythmia. The importance of Cav3 changes in HF and downstream microdomain dysregulation may have important implications for arrhythmia generation.

## Author Contributions

RV contributed to writing, figure production, and editing. LR contributed to manuscript writing and editing. LE contributed to project conceptualization, writing, figure production, and editing.

## Conflict of Interest Statement

The authors declare that the research was conducted in the absence of any commercial or financial relationships that could be construed as a potential conflict of interest.
